# *Klebsiella pneumoniae* liver abscess in diabetic patients: association of glycemic control with the clinical characteristics

**DOI:** 10.1186/1471-2334-13-56

**Published:** 2013-01-30

**Authors:** Yi-Tsung Lin, Fu-Der Wang, Ping-Feng Wu, Chang-Phone Fung

**Affiliations:** 1 Division of Infectious Diseases, Department of Medicine, Taipei Veterans General Hospital, No. 201, Sec. 2, Shih-Pai Road, 112, Taipei, Taiwan; 2 School of Medicine, National Yang-Ming University, Taipei, Taiwan

## Abstract

**Background:**

*Klebsiella pneumoniae* liver abscess (KPLA) has been reported with increasing frequency in East Asian countries in the past 3 decades, especially in Taiwan and Korea. Diabetes is a well-known risk factor for KPLA and highly associated with septic metastatic complications from KPLA. We investigated the association of glycemic control in diabetic patients with the clinical characteristics of KPLA in Taiwan.

**Methods:**

Adult diabetic patients with KPLA were identified retrospectively in a medical center from January 2007 to January 2012. Clinical characteristics were compared among patients with different levels of current hemoglobin A1c (HbA_1c_). Risk factors for metastatic infection from KPLA were analyzed.

**Results:**

Patients with uncontrolled glycemia (HbA_1c_ ≥ 7%) were significantly younger than those with controlled glycemia (HbA_1c_ < 7%). Patients with uncontrolled glycemia had the trend to have a higher rate of gas-forming liver abscess, cryptogenic liver abscess, and metastatic infection than those with controlled glycemia. Cryptogenic liver abscess and metastatic infection were more common in the poor glycemic control group (HbA_1c_ value >; 10%) after adjustment with age. HbA_1c_ level and abscess < 5 cm were independent risk factors for metastatic complications from KPLA.

**Conclusions:**

Glycemic control in diabetic patients played an essential role in the clinical characteristics of KPLA, especially in metastatic complications from KPLA.

## Background

*Klebsiella pneumoniae* liver abscess (KPLA) has been reported with increasing frequency in East Asian countries in the past 3 decades, especially in Taiwan and Korea [1-7]. *K*. *pneumoniae* is the dominant cause of pyogenic liver abscesses in Taiwan and has contributed to the endemic feature of the disease in Taiwan [8-10]. Since 1986, many researchers in Taiwan and several other areas have noted the distinctive syndrome of KPLA, complicated by bacteremia, sepsis, and metastatic infection of brain, eyes, lungs and other organs, especially in patients with diabetes [2,5,6,11-15]. The mortality rate of KPLA has decreased in recent years in Taiwan [6,8]; nevertheless, despite aggressive therapy, the outcomes of these patients frequently involve catastrophic disability. Our recent study also has indicated that catastrophic disability due to ocular or neurological complications from KPLA could lead to poor long-term prognosis [10].

Patients with diabetes are at increased risk for common infection due to impaired host defense mechanisms [16]. Diabetes is a well-known risk factor for pyogenic liver abscess [17] and highly associated with KPLA [5,18,19]. One animal study of KPLA has suggested that diabetes might provide a specialized environment that allows *K*. *pneumoniae* strains to disseminate from the intestines into the blood [20]. In addition, diabetes is the most common underlying disease among patients with septic metastatic complications from KPLA [12,13,19,21], and predisposes patients with KPLA to develop septic metastasis [22]. Compared with the monomicrobial cryptogenic noninvasive KPLA, cryptogenic KPLA with septic metastasis is characterized by an ~20-fold increased association with diabetes [23].

Previous studies have compared the characteristics of liver abscess patients with and without diabetes [17,18,24]. The influence of diabetes on KPLA has also been described [1]. However, no research is available on the impact of glycemic control on the characteristics of KPLA in diabetic patients. To monitor glycemic control, hemoglobin A1c (HbA_1c_) gives an estimate of average blood glucose during the preceding 3 months, and is widely accepted as the primary indicator of the level of glycemic control for optimal management of diabetes [25].

Therefore, we conducted this study to investigate whether glycemic control assessed by HbA_1c_ affected the clinical characteristics of diabetic patients with KPLA.

## Methods

### Study design, patient populations, and data collection

This study was a retrospective cohort analysis of consecutive diabetic patients with KPLA at Taipei Veterans General Hospital, a tertiary medical center with a 2900-bed capacity. Those patients with KPLA were identified by reviewing culture records from the Department of Microbiology from January 2007 to January 2012. We excluded patients aged < 20 years. Age, sex, underlying diseases, clinical presentations, laboratory findings, including images, and management were collected. Assessment of blood glucose control was based on HbA_1c_ levels at the time of infection or as close to the time of infection as possible, within one month before this episode of KPLA. The major outcome measurements included intensive care unit admission within 48 hours, length of hospitalization, and mortality. Septic metastatic infection is highly associated with diabetic patients with KPLA; therefore, we analyzed the risk factors among these patients. This study was approved by Institutional Review Board of Taipei Veterans General Hospital.

### Definitions

An episode of KPLA was defined as the presence of ≥ 1 liver abscess, detected by sonography or computed tomography, and culture-confirmed *K*. *pneumoniae* isolated from an abscess or blood. Only the first episode of KPLA in a particular patient, diagnosed at our hospital during the period of study, was included. For the diagnosis of diabetes, the American Diabetes Association diagnostic criteria were used [25]. Patients with diabetes were defined as those with a history of either type 1 or type 2 diabetes and/or those taking either insulin and/or oral hypoglycemic agents. If diabetes was diagnosed during the patient’s treatment for this episode of KPLA, newly diagnosed diabetes was recorded. Chronic kidney disease was indicated by a creatinine level ≥ 2.0 mg/dL. Chronic lung disease was defined as chronic obstructive pulmonary diseases, bronchiectasis, or any structural lung diseases with the exception of bronchogenic carcinoma. Cryptogenic origin of infection was defined as that in which no obvious extrahepatic source of infection could be identified [22]; biliary tract origin was defined if clinical features of cholecystitis/cholangitis or extrahepatic biliary ductal abnormalities were identified on radiographic images. Multiple abscesses mean >; 2 abscess in liver parenchyma confirmed by imaging. Metastatic infection was defined as a distant site of infection isolated with the same pathogen as the pyogenic liver abscess (*K*. *pneumoniae*).

### Microbiology laboratory procedures

The VITEK 2 system (bioMérieux, Marcy l'Etoile, France) was used to confirm bacterial identifications among the available isolates. *magA* is the serotype K1 wzy allele and was determined among the *K*. *pneumonaie* isolates as described previously [6].

### Statistical analysis

We performed a 2-tailed χ^2^ test or Fisher’s exact test (for contingency data) and Student’s *t* test or ANOVA method (for continuous data) to compare the groups with different HbA_1c_ levels. A *P* value < 0.05 was considered statistically significant; all probabilities were 2-tailed. Cochran-Mantel-Haenszel test with median age as the stratum was used to analyze the association between glycemic control and other factors. To identify possible independent predictors for metastatic infection, multivariate analysis was undertaken using logistic regression to adjust for the presence of confounding variables. Age, sex and all potential predictors with *p* ≦ 0.1 on univariate analysis were considered for inclusion in the multivariate model. All statistical analyses were performed with SPSS version 17.0 for Windows.

## Results

### Comparison of clinical characteristics of KPLA in diabetic patients with controlled or uncontrolled glycemia

During the study period, 108 diabetic patients from all of the 221 KPLA cases were identified. We excluded six patients due to lack of HbA_1c_. The remaining 102 patients all had type 2 diabetes. HbA_1c_ level < 7% is generally considered as the target of glycemic control for diabetic patients [25,26]. We compared patients with controlled glycemia (HbA_1c_ < 7%) and uncontrolled glycemia (HbA_1c_ ≥ 7%). The level of HbA_1c_ in the group with uncontrolled glycemia was significantly higher than that in the group with controlled glycemia (10.0 ± 2.1 vs 6.5 ± 0.3%, *p* < 0.001).

Table 1 showed the clinical features between these two groups. Patients with uncontrolled glycemia were younger (62.8 ± 14.9 vs 70.6 ± 9.6 years, *p* = 0.017) than those with controlled glycemia. Patients with poor glycemic control had the trend to have a higher rate of cryptogenic liver abscess (92.8 vs 78. 9%, *p* = 0.087), gas-forming liver abscess (14.5 vs 0%, *p* = 0.116), and metastatic infection (14.5 vs 0%, *p* = 0.116) than those with controlled glycemia. The underlying disease did not differ between the two groups. Most of the lesions were located in the right lobe, but the characteristics of abscess did not differ significantly between the two groups. No mortality was identified in the group with controlled glycemia. The treatment strategy did not differ among the groups with different HbA_1c_ levels. Length of stay in hospital and intensive care unit admission rate did not differ significantly between the two groups. As the age is significantly different between the groups, we compared all the characteristics after adjustment with age, which showed the consistent results. Only 41 isolates of *K*. *pneumonaie* were available for microbiological analysis, and we found that 21 isolates were positive for *magA*. The distribution of *magA* in *K*. *pneumoniae* isolates did not differ between controlled and uncontrolled groups (66.7% vs 46.9%, *p* = 0.454).

**Table 1:** Baseline characteristics, clinical presentation, and outcome of diabetic patients with controlled or uncontrolled glycemia

	**Controlled glycemia (n = 19)**	**Uncontrolled glycemia (n = 83)**	***p *value**	***p *value, (adjusted age)**
Male	12 (63.2)	59 (71.1)	0.498	0.952
Age in years	70.6 ± 9.6	62.8 ± 14.9	0.017	
Underlying diseases				
Malignancy	1 (5.6)	10 (12.7)	0.683	0.367
Alcoholism	1 (5.3)	4 (4.8)	1.000	0.655
Chronic kidney disease	1 (5.6)	8 (10.3)	1.000	0.311
Liver cirrhosis	0 (0)	2 (2.4)	1.000	0.494
Congestive heart failure	0 (0)	2 (2.4)	1.000	0.392
Chronic lung disease	0 (0)	1 (1.2)	1.000	0.549
Newly diagnosed diabetes	4 (21.1)	21 (25.3)	1.000	0.995
Origin				
Cryptogenic	15 (78.9)	77 (92.8)	0.087	0.090
Biliary tract origin	1 (5.3)	5 (6.0)	1.000	0.785
Abscess locations				
Right lobe	11 (57.9)	44 (53.0)	0.791	0.407
Left lobe	5 (26.3)	19 (22.9)		
Both lobes	3 (15.8)	20 (24.1)		
Abscess size				
< 5 cm	9 (47.4)	36 (43.4)	1.000	0.707
5 ~ 10 cm	9 (47.4)	39 (47.0)		
>; 10 cm	1 (5.3)	8 (9.6)		
Gas forming	0 (0)	12 (14.5)	0.116	0.126
Multiple abscesses	8 (42.1)	29 (34.9)	0.558	0.791
Initial laboratory value				
Leukocyte count, × 10^3^/μL	12.4 ± 3.8	13.5 ± 6.0	0.165	0.417
Platelet, × 10^3^/μL	195 ± 87	229 ± 121	0.145	0.442
C-reactive protein, mg/dL	18.8 ± 9.2	20.0 ± 9.8	0.487	0.779
Glucose, mg/dL	193 ± 76	318 ± 168	0.007	0.004
Treatment				
Antibiotics + drainage	16 (84.2)	69 (83.1)	1.000	0.894
Antibiotics + operation	1 (5.3)	3 (3.6)	0.568	0.525
Antibiotics only	2 (10.5)	11 (13.4)	1.000	0.557
Metastatic infections	0 (0)	12 (14.5)	0.116	0.077
Outcome				
Hospitalization days	26.2 ± 12.1	28.1 ± 19.0	0.692	0.428
Intensive care unit admission	2 (10.5)	12 (14.5)	1.000	0.849
Mortality	0 (0)	3 (3.6)	1.000	0.359

Data are presented as mean ± SD or frequency with percentage (%).

### Comparison of clinical characteristics in KPLA diabetic patients according to different HbA_1c_ levels

To analyze further the impact of glycemic control on the clinical characteristics of KPLA, we divided the patients into three groups according to HbA_1c_ level: < 7%, 7–10% and >; 10%, as shown in Table 2. A HbA_1c_ level >; 10% suggested poorly controlled diabetes [25]. The level of HbA_1c_ was significantly different in the three groups (6.5 ± 0.3 vs 8.4 ± 0.9 vs 11.8 ± 1.6%, *p* < 0.001). Patients with HbA_1c_ >; 10% were significantly younger (*p* < 0.001) than the other two groups. Newly diagnosed diabetes, cryptogenic origin abscess, and metastatic infection were more common in the group of HbA_1c_ level >; 10%. Cryptogenic origin abscess and metastatic infection were still more common in the group of HbA_1c_ level >; 10% after adjustment with age.

**Table 2:** **Baseline characteristics, clinical presentation, and outcome of diabetic patients with KPLA according to different HbA**_**1c **_**levels**

**Factor**	**HbA**_**1c**_** < 7% (n = 19)**	**HbA**_**1c**_** = 7 ~ 10% (n = 44)**	**HbA**_**1c**_** >; 10% (n = 39)**	***p *value**	***p *value, (adjusted age)**
Male	12 (63.2)	30 (68.2)	29 (74.4)	0.660	0.999
Age in years	71.3 ± 9.9	68.2 ± 11.4	56.3 ±15.8	<0.001	
Underlying diseases					
Malignancy	1 (5.6)	8 (20.0)	2 (5.1)	0.100	0.560
Alcoholism	1 (5.3)	0 (0)	4 (10.3)	0.081	0.378
Chronic kidney disease	1 (5.6)	7 (17.5)	1 (2.6)	0.067	0.889
Liver cirrhosis	0 (0)	2 (4.5)	0 (0)	0.667	0.676
Congestive heart failure	0 (0)	2 (4.5)	0 (0)	0.667	0.970
Chronic lung disease	0 (0)	1 (2.3)	0 (0)	1.000	0.979
Newly diagnosed diabetes	4 (21.1)	6 (13.6)	15 (38.5)	0.028	0.146
Origin					
Cryptogenic	15 (78.9)	38 (86.4)	39 (100.0)	0.008	0.008
Biliary tract origin	1 (5.3)	5 (11.4)	0 (0)	0.068	0.287
Abscess locations					
Right lobe	11 (57.9)	23 (52.3)	21 (53.8)	0.442	0.214
Left lobe	5 (26.3)	13 (29.5)	6 (15.4)		
Both lobes	3 (15.8)	8 (18.2)	12 (30.8)		
Abscess size					
< 5 cm	9 (47.4)	18 (40.9)	18 (46.2)	0.962	0.829
5 ~ 10 cm	9 (47.4)	21 (47.7)	18 (46.2)		
>; 10 cm	1 (5.3)	5 (11.4)	3 (7.7)		
Gas forming	0 (0)	6 (13.6)	6 (15.4)	0.218	0.317
Multiple abscesses	8 (42.1)	12 (27.3)	17 (43.6)	0.256	0.308
Initial laboratory value					
Leukocyte count, × 10^3^/μL	12.4 ± 3.8	14.4 ± 5.6	12.4 ± 6.2	0.168	0.225
Platelet, × 10^3^/μL	195 ± 87	222 ± 102	237 ± 140	0.380	0.734
C-reactive protein, mg/dL	18.8 ± 9.2	19.3 ± 9.6	20.9 ± 10.1	0.539	0.826
Glucose, mg/dL	193 ± 76	270 ± 152	371 ± 170	<0.001	<0.001
Treatment					
Antibiotics + drainage	16 (84.2)	37 (84.1)	32 (82.1)	1.000	0.779
Antibiotics + operation	1 (5.3)	2 (4.5)	1 (2.6)	1.000	0.345
Antibiotics only	2 (10.5)	6 (13.6)	5 (13.2)	1.000	0.524
Metastatic infections,	0 (0)	3 (6.8)	9 (23.1)	0.021	0.004
Outcome					
Hospitalization days	26.3 ± 15.7	31.9 ± 23.2	24.9 ± 12.5	0.201	0.265
ICU admission	2 (10.5)	5 (11.4)	7 (17.9)	0.695	0.605
Mortality	0 (0)	1 (2.3)	2 (5.1)	0.785	0.181

Data are presented as mean ± SD or frequency with percentage (%).

### Risk factors for metastatic infections from KPLA in diabetic patients

The rate of metastatic infection was 11.8% in diabetic KPLA (n = 12), and the commonest infectious site was the eyes (n = 3) and lungs (n = 3), followed by the spleen (n = 2), kidneys (n = 2), prostate (n = 1), and muscle over the left thigh (n = 1). Two patients with metastatic infection died and the mortality rate was significantly higher than that without metastatic infection (16.7 vs 1.1%, *p* = 0.036). Table 3 showed the risk factors metastatic infection from KPLA. Logistic regression analysis revealed that HbA_1c_ level (odds ratio [OR], 1.50; 95% confidence interval [CI], 1.01-2.25; *p* = 0.047) abscess size < 5 cm (OR, 4.72; 95% CI, 1.09–20.52; *p* = 0.038) were the independent predictors of metastatic infection.

**Table 3:** Risk factors for metastatic infection of KPLA in diabetic patients

	**No metastatic infection (n = 90)**	**Metastatic infection (n = 12)**	**Univariate analysis**	**Multivariate analysis**	
**Factor**			***p *value**	**Odds Ratio (95% CI)**	***p *value**
Male	63 (70.0)	8 (66.7)	0.814	0.76 (0.16–3.59)	0.733
Age in years	64.4 ± 14.2	63.2 ± 16.0	0.779	1.02 (0.97–1.08)	0.430
Underlying diseases					
Malignancy	10 (11.8)	1 (8.3)	0.772	–	–
Alcoholism	4 (4.8)	1 (8.3)	0.564	–	–
Chronic kidney disease	9 (10.6)	0 (0)	0.999	–	–
Liver cirrhosis	2 (2.2)	0 (0)	0.999	–	–
Congestive heart failure	2 (2.2)	0 (0)	0.999	–	–
Chronic lung disease	1 (1.1)	0 (0)	1.000	–	–
Newly diagnosed diabetes	21 (23.3)	4 (33.3)	0.453	–	–
HbA_1c_	9.1 ± 2.3	11.0 ± 2.3	0.015	1.50 (1.01–2.25)	0.047
Cryptogenic origin	80 (88.9)	12 (100)	0.999	–	–
Abscess locations in both lobes	18 (20.0)	5 (41.7)	0.102	1.48 (0.29–7.60)	0.646
Abscess size < 5 cm	36 (40)	9 (75)	0.032	4.72 (1.09–20.52)	0.038
Gas forming	11 (12.2)	1 (8.3)	0.693	–	–
Multiple abscesses	29 (32.2)	8 (66.7)	0.028	3.03 (0.62–14.74)	0.170
Initial laboratory value					
Leucocyte count, × 10^3^/μL	13.2 ± 5.5	13.9 ± 6.8	0.653	–	–
Platelet, × 10^3^/μL	225 ± 117	209 ± 113	0.654	–	–
C-reactive protein, mg/dL	19.5 ± 9.6	22.3 ± 9.6	0.339	–	–
Glucose, mg/dL	284 ± 150	380 ± 229	0.071	1.00 (0.99–1.00)	0.728

Data are presented as mean ± SD orf frequency with percentage (%).

## Discussion

It is believed that several aspects of immunity are altered in patients with diabetes. For example, polymorphonuclear leukocyte function is depressed; leukocyte adherence, chemotaxis and phagocytosis may be affected; and antioxidant systems involved in bactericidal activity may also be impaired. Although these *in vitro* findings have not yet been fully confirmed in clinical studies, there is evidence that improving glycemic control in patients improves immune function [27]. Kornum et al. found that an elevated HbA_1c_ predicted increased risk for all-cause community-acquired pneumonia among patients with diabetes [28]. In diabetic patients with sepsis, one study has demonstrated that HbA_1c_ is an independent prognostic factor for hospital mortality and length of stay [29]. Regarding the issue of glycemic control and KPLA, only one case series from Taiwan analyzing 6 cases of recurrent KPLA has been reported in the literature. Five of the six patients had diabetes with poor glycemic control, and four had HbA_1c_ >; 9% (range: 9.2–17.5%) [30]. The current study is, to the best of our knowledge, the first to investigate the effect of glycemic control on characteristics of KPLA in diabetic patients.

In our study, we found that patients with uncontrolled glycemia tended to be younger, and had the trend to have a higher rate of cryptogenic liver abscess, gas-forming liver abscess, and metastatic infection than those with controlled glycemia, although these differences did not reach significance. Young age, newly diagnosed diabetes, cryptogenic liver abscess and metastatic infection were more common in the poor glycemic control group (HbA_1c_ value >; 10%). Cryptogenic invasive KPLA is frequently associated with diabetes [23]; therefore, we further established that it was highly linked to diabetic patients with high level of HbA_1c_. The finding that younger patients with KPLA are prone to have uncontrolled glycemia has never been reported, in part due to the small sample sizes of studies reported in the literature [6,13].

Lin et al. have analyzed the effect of glycemic control of type 2 diabetes on neutrophil phagocytosis of serotype K1/K2 *K*. *pneumoniae* isolates. This *in vitro* study suggests that strict metabolic control may improve the neutrophil phagocytosis of K1/K2 *K*. *pneumoniae* in patients with type 2 diabetes [31]. In the current study focusing on diabetic patients, we not only found that the rate of metastatic infection was more common in patients with poor glycemic control, but also that HbA_1c_ level was an independent risk factor for metastatic infection. It is also notable that there was no mortality, metastatic infection or gas-forming abscess in the group with controlled glycemia. The previous *in vitro* study by Lin et al. may support our clinical findings, and we suggest that HbA_1c_ level is detrimental in KPLA, and controlled glycemia may prevent the development of serious metastatic complications.

Previous case reports have demonstrated that diagnosis of diabetes may come to light because of KPLA [32,33]. It is notable that around 25% of cases were newly recognized diabetes in the current study despite improvement in diagnosis and awareness in recent years, and the existence of a comprehensive national health insurance program in Taiwan that optimizes access to medical care. Due to unrecognized diabetes complicated with infection, extreme hyperglycemia, even hyperglycemic hyperosmolar state or diabetic ketoacidosis, may occur. Physicians should not ignore the underlying diabetes in patients with KPLA in clinical practice. One particularly interesting finding in our study was that liver abscess < 5 cm in diabetic patients was independently associated with metastatic infection, which has not been reported in the literature. It suggested that we should not overlook the risk of complication from small sized liver abscess in diabetic patients.

We acknowledge some limitations in our study that deserve mention. First, the data were collected retrospectively from medical records. Second, participants were recruited from a medical center in Northern Taiwan, and therefore we could not evaluate possible regional variations. Further analyses should be conducted in other regions and countries to confirm our findings. Thirdly, our results were also limited by the incomplete collection of HbA_1c_ data. Finally, the isolates were not collected for further analysis, such as virulence factors or clonal types, in the current study. Despite these limitations, our study is believed to be the first to discuss the characteristics of KPLA due to the different level of glycemic control, which sheds further light on the association between diabetes and KPLA, and our results may partly elucidate the role of host factors in the pathogenesis of KPLA.

## Conclusion

Patients with uncontrolled glycemia were significantly younger than those with controlled glycemia. Patients with uncontrolled glycemia had the trend to have a higher rate of gas-forming liver abscess, cryptogenic liver abscess and metastatic infection than those with controlled glycemia. Cryptogenic liver abscess and metastatic infection were more common in the poor glycemic control group (HbA_1c_ value >; 10%). HbA_1c_ level and abscess < 5 cm were independent risk factors for metastatic complications from KPLA. Therefore, glycemic control in diabetic patients plays an essential role in the clinical features of KPLA, especially in metastatic complications from KPLA.

## Abbreviations

KPLA: *Klebsiella pneumoniae* liver abscess; 95% CI: 95% confidence interval; OR: Odds ratio; HbA_1c_: Hemoglobin A1c.

## Competing interests

All authors declare that they have no conflict of interest.

## Authors’ contributions

YTL conceived of the study, and participated in its design and coordination. YTL, FDW, PFW and CPF performed the laboratory experiments, reviewed and collected the data. YTL analyzed and interpreted the data. YTL and PFW drafted the manuscript. FDW and CPF reviewed the manuscript. All authors read and approved the final manuscript.

## Pre-publication history

The pre-publication history for this paper can be accessed here:

http://www.biomedcentral.com/1471-2334/13/56/prepub
